# Effectiveness of strontium/silver-based titanium surface coatings in improving antibacterial and osteogenic implant characteristics: a systematic review of *in-vitro* studies

**DOI:** 10.3389/fbioe.2024.1346426

**Published:** 2024-02-29

**Authors:** Marjan Kheirmand-Parizi, Katharina Doll-Nikutta, Amit Gaikwad, Hannah Denis, Meike Stiesch

**Affiliations:** ^1^ Department of Prosthetic Dentistry and Biomedical Materials Science, Hannover Medical School, Hannover, Germany; ^2^ Lower Saxony Center for Biomedical Engineering, Implant Research and Development (NIFE), Hannover, Germany

**Keywords:** antibacterial, osteogenic, silver, strontium, titanium

## Abstract

**Introduction:** Due to the high incidence of implant failures, dual functionalization of titanium surfaces with antibacterial and osteogenic agents, like silver (Ag) and strontium (Sr), has gained significant attention in recent years. However, so far, the combined antibacterial and osteoinductive effectiveness of Ag/Sr-based titanium surface coatings has only been analyzed in individual studies.

**Methods:** This systematic review aims to evaluate the existing scientific literature regarding the PICOS question “Does dual incorporation of strontium/silver enhances the osteogenic and anti-bacterial characteristics of Ti surfaces *in vitro*?”. As a result of a web-based search adhering to the PRISMA Guidelines using three electronic databases (PubMed, Scopus, and Web of Science) until March 31, 2023, a total of 69 publications were identified as potentially relevant and 17 of which were considered appropriate for inclusion into this review.

**Results and Discussion:** In all included publications, the use of Sr/Ag combination showed enhanced osteogenic and antibacterial effects, either alone or in combination with other agents. Moreover, the combination of Sr and Ag shows potential to synergistically enhance these effects. Nevertheless, further studies need to validate these findings under clinically more relevant conditions and evaluate the mechanism of antimicrobial and osteogenic activity of Sr/Ag combination.

## 1 Introduction

Titanium (Ti) and its alloys, presenting superior biocompatibility and excellent corrosion resistance, are often the preferred choice for implant materials (Hanawa, 2019). Despite their high success rates, certain risk factors make it difficult to completely avoid implant failures. Approximately 11% of dental implants and 10% of orthopedic implants fail and need to be removed due to undesired complications (Kurtz et al., 2005; Souza et al., 2018). Implant infections were reported in 28.8% of patients with dental implants (functional time ≥5 years) and 30% of patients following orthopedic internal fixation (Dreyer et al., 2018; Kuehl et al., 2019). This aids in damage of peri-implant tissues affecting direct bone-to-implant contact thus resulting in implant failure (Thomas and Puleo, 2011). Moreover, aseptic loosening occurred due to tiny gaps of implant–bone interface and likewise reduces long-term stability of orthopedic implants (Sundfeldt et al., 2006). Hence, the need for establishment of implant surface with dual properties including osteogenic and antibacterial is essential for the long-term survival and prevention of implant associated infections (Nguyen-Hieu et al., 2012; Xue et al., 2020; Lu et al., 2021).

Different dual functionalization strategies have been adopted on Ti surfaces (Shi et al., 2008; Raphel et al., 2016; Lu et al., 2021). These include combed strategies for improving tissue integration such as surface modification or coating hydroxyapatite, growth factors, metal ions, etc. with antimicrobial strategies such as metal ions, antimicrobial drugs, polymers/copolymers, antimicrobial peptides (AMP), nitride, UV and laser-activatable surfaces, etc. (Grischke et al., 2016; Raphel et al., 2016; Lu et al., 2021). Silver (Ag) as one of the most effective antibacterial metals was used in multiple strategies resulting in bio-functionalized antibacterial Ti surfaces (Raphel et al., 2016; Lu et al., 2021; Sui et al., 2022). Ag-functionalized implant surfaces can significantly decrease bacterial adhesion and biofilm formation by disrupting the bacterial wall/membrane, signal transduction pathways, intracellular penetration, and inducing oxidative stress (Tripathi and Goshisht, 2022). However, the use of Ag in this context has limitations due to its cytotoxic effects on surrounding tissues, which must be taken into account when considering clinical applications (Tripathi and Goshisht, 2022).

In order to enhance osseointegration properties, several Ti coatings have incorporated strontium (Sr). Sr as an alkaline metal has already been used clinically as a therapeutic reagent for osteoporosis in the form of strontium ranelate (Marx et al., 2020). Based on a systematic review on animal models, bone-to-implant contact (BIC) of Ti implants modified with Sr was significantly higher than for unmodified implants (Shi et al., 2017). The presence of Sr ions promotes osteoblast proliferation and differentiation, while inhibiting the differentiation of osteoclasts (Marx et al., 2020). Additionally, Sr-functionalized Ti implants showed a limited antimicrobial effect for a short period of time (Alshammari et al., 2021).

Thus, combined application of biofunctional elements, such as Sr and Ag, presents a multifunctional strategy that could simultaneously promote bone formation and prevent bacterial colonization, thereby promoting the success rate of the titanium implants (Xu et al., 2020). For instance, Sr/Ag loaded nanotubular structures on Ti surface demonstrated long-lasting and remarkable anti-adhesive and antibacterial characteristics against various types of bacteria. Moreover, the coating accelerated the healing of bone defects and enhanced the expression of osteoblastic phenotype *in vivo* and *in vitro* respectively (Cheng et al., 2016). Our recent study on the biological potential of Sr/Ag combination showed that Sr may not only partially compensate the cytoxicity of Ag facilitating bone formation but also exhibited a synergistic antibacterial effect when combined with Ag (Parizi et al., 2022). Nevertheless, these are results of individual studies. So far, even as there exist general reviews on dual functionalization strategies (Xue et al., 2020), Sr/Ag modified Ti surfaces have not been assessed comprehensively across studies for their combined antibacterial and osteoinductive effects. Thus, the purpose of this review was to systematically analyze the existing literature using the Preferred Reporting Items for Systematic Review and Meta-Analyses (PRISMA) guidelines (Page et al., 2021) regarding the development and efficacy of Sr/Ag functionalized Ti surfaces toward dual antibacterial and osteoinductive properties. Additionally, a second focus was put on the applied experimental methods to analyze both properties. The meta-analytical overview generated in this review should support future researchers in implementing effective approaches to develop and analyze dual-functionalized implant surfaces.

## 2 Methods

### 2.1 Protocol development

This systematic review was conducted in accordance with the Preferred Reporting Items for Systematic Review and Meta-Analyses (PRISMA) guidelines. The following PICOS elements were defined:

(P) Population: Ti implants
(I) Intervention: Ti implants functionalized with Sr and Ag
(C) Control: Ti implants that do not use Sr/Ag combinations
(O) Outcomes: Antibacterial activity and osteogenic characteristics
(S): Study design: *In-vitro* studies

The following research question was addressed based on the PICOS element: “Does dual incorporation of strontium/silver enhances the osteogenic and anti-bacterial characteristics of Ti surfaces *in vitro*?”

### 2.2 Search strategy

A comprehensive literature search was performed across PubMed, Scopus, and Web of Science electronic databases using specific keywords and MeSH terms. The following terms were included: “Osseointegration” OR “osteogenic differentiation” OR “bone regeneration” OR “osseointegration” OR “Osteoconductive” OR “Osteoinductive” OR “osteoblast differentiation” OR “osteogenic gene expression” OR “mineralization” AND “anti-bacterial activity” OR “antimicrobial” OR “anti-infection” OR “antiBiofilm” OR “bactericidal” OR “bacteriostatic” AND “Titanium” OR “Ti implant” OR “Ti sheet” OR “titanium disk” OR “titanium substrate” OR “titanium plate” AND “Silver” OR “silver nanoparticle” OR “Ag” OR “AgNPs” OR “Ag ions” OR “Ag nanoparticles” AND “strontium” OR “Sr.” Manual searches were performed in peer-reviewed journal in the field of implantology. In addition, references of included and excluded studies were scrutinized to identify additional studies. The last search was performed on 31 March 2023.

### 2.3 Eligibility criteria and study selection process

After a comprehensive search, studies were included based on the eligibility criteria as presented in Table 1. Searches were performed by two review authors (M.P. and H.D.) independently to identify the potential studies. Any disagreements regarding the inclusion of studies were sorted with discussion. Any further discrepancy was resolved with the opinion of a third reviewer (K.D-N.) until reaching a unanimous decision.

**TABLE 1:** Inclusion and exclusion criteria for study selection.

Inclusion criteria	Exclusion criteria
*In-vitro* studies evaluating antimicrobial and osteogenic potential of strontium/silver functionalized Ti implants	Case reports, Case series sample sizes under 10, Conference papers, Review articles, Author responses, Hypotheses
Studies with full text availability	Missing either *in vitro* microbial tests OR *in vitro* cell differentiation tests
Studies published in English language only	Missing using silver/strontium
	Grey literature
	Missing using Ti as substrate
	Studies published in Language other than English
	Only *in-vivo* study

### 2.4 Risk of bias

The risk of bias assessment was performed using a tool as reported in a previous study (Mendes et al., 2022) with few modifications. These modifications were implemented due to the categories of studies included in the present review. The tool presents 9 domains including clearly stated aims/objectives, presence of adequate control, ion release analysis, standardization in sample production process, sample characterization, assessment of antibacterial and osteogenic methods, observer blinding and adequate statistical analyses. Each of these domains were presented with subsequent answer ‘reported’ or ‘not reported’. If the domains were adequately reported, then the study was presented with score 1 and score 0 in situation if the data was missing. Overall, the study was rated with ‘high’ risk if scored less than 3, ‘moderate’ if scored 4 to 6 and low if scored 7 to 9. The risk of bias assessment was performed by two review authors (M.P. and A.G.) independently. The disparity between the decisions was resolved with profound discussion until a consensus was reached or by discussion with third review author (K.D-N.).

### 2.5 Data extraction and statistical analyses

Following data was extracted of all included studies: author, year of publication, surface modification, fabrication method, bacteria tested, antibacterial assay, intergroup comparisons, cells tested, osteogenic markers and their detecting assays, and conclusion. All data from the included studies was analyzed qualitatively. Because of the high heterogeneity reported in the included studies with respect to the outcomes and the methodology, meta-analysis was not performed.

## 3 Results

### 3.1 Search results

**FIGURE 1:**
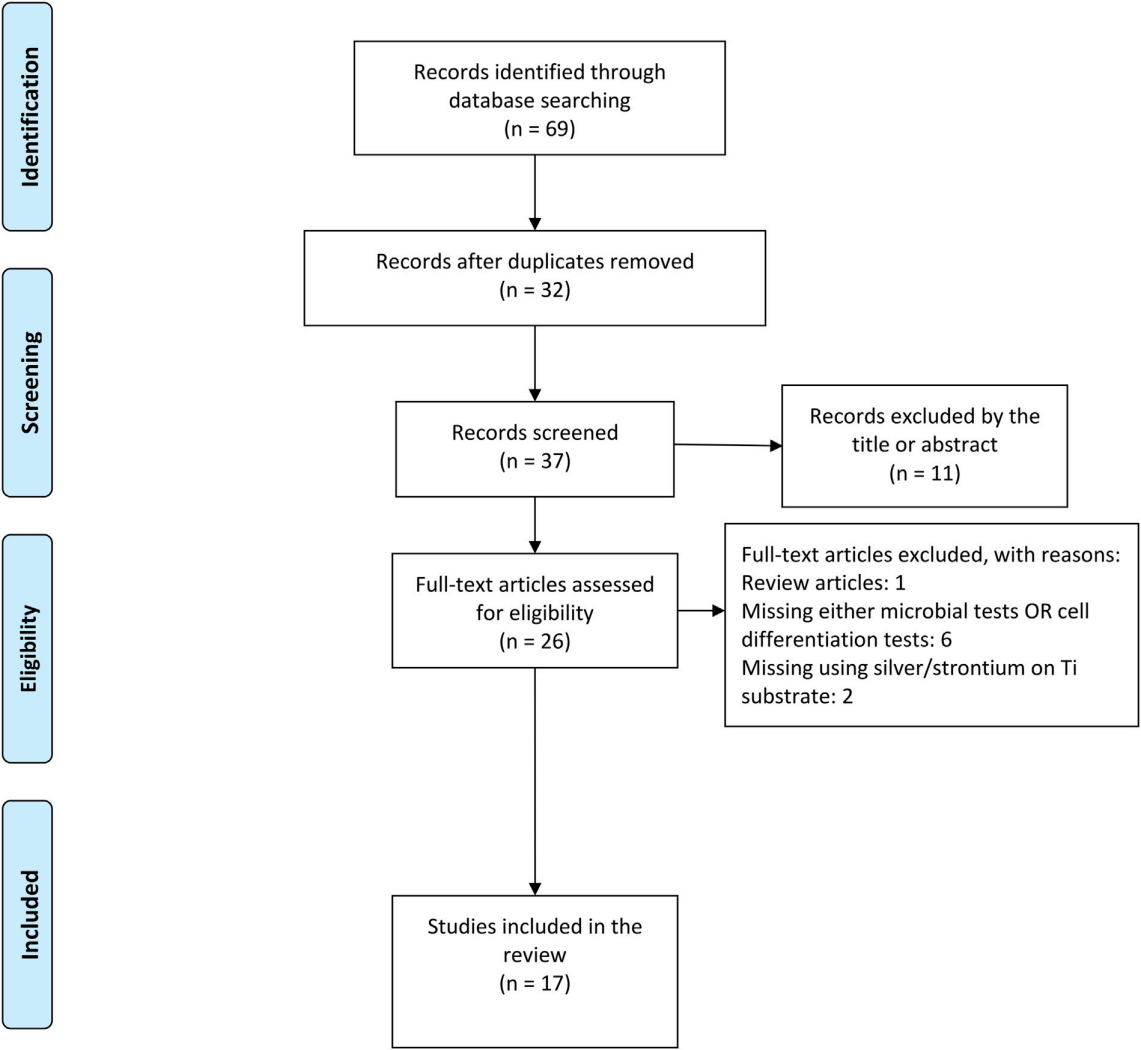
PRISMA flow chart showing the study selection process on strontium/silver-based titanium surface for antibacterial and osteogenic characteristics.

A total number of 69 articles were initially identified as a direct result of electronic PICOS-based search in three databases. The process of exclusion and inclusion of potentially relevant articles is illustrated in a flow chart (Figure 1). First, 32 duplicates were removed and out of the remaining 37 articles, 26 were considered potentially eligible based on the title and/or abstract. Screening of full-text manuscripts led to further exclusions of 9 publications, as they failed the study’s selection criteria (Table 1). Consequently, 17 articles were included in the present review.

### 3.2 Surface functionalization and release properties

Different surface modification methods have been used to functionalize Ti surfaces with Ag and Sr. Among the included studies, eight used hydrothermal treatment, five electrodeposition, three micro-arc oxidation or plasma electrolytic oxidation, and remaining one used plasma spray technique to coat one or more than one chemical on the Ti substrates (Table 2). All included studies had physically modified Ti surface topography. Microstructures and porosities were applied either separately or accompanied by the result of chemical coatings. Majority of (70%) included studies presented nano-level modifications in the forms of nanostructured Ti (NT), TiO_2_ nanotubes (TNT) and nanoparticle (NP) structures (Cheng et al., 2016; Chen et al., 2017; Geng et al., 2017; Huang et al., 2017; Li et al., 2019; Pan et al., 2020; van Hengel et al., 2020; Zhang et al., 2020; Wang et al., 2021a; Huang et al., 2022; Yao et al., 2022; Bian et al., 2023). Additionally, functionalized Ti substrates were coated either only with Sr/Ag or in combination with other coatings including hydroxyapatite (HA), calcium (Ca), silk (Si), fibroin (F), manganese (Mn) and graphene oxide (GO) (Table 2). Among these, HA was the most frequently used additional coating and presented in seven of included studies. Despite the parameters extracted for the following meta-analysis, all included studies performed initial physical and chemical characterizations of their coatings, regarding, e.g., surface topography, chemical composition or wettability. Also, all but one study analyzed the release of silver and/or strontium ions (Table 2). Most release curves were recorded for 14 or 21 days and revealed an almost linear zero order release (50%). Four studies observed a burst release within a maximum of 4 days (Cheng et al., 2016; Pan et al., 2020; van Hengel et al., 2020; Zhang et al., 2021), whereas three studies showed steadily decreasing first order releases (Chen et al., 2017; Wang et al., 2021a; Bian et al., 2023). Even though only 50% of studies compared the release patterns of different coatings, these could mainly identify no differences (Fielding et al., 2012; Chen et al., 2017; Geng et al., 2017; Qiao et al., 2019; Huang et al., 2022; Yao et al., 2022) and only in two cases a slower release of the Ag/Sr combined coating (van Hengel et al., 2020; Wang et al., 2021a).

**TABLE 2:** Surface coatings and modification approaches.

Author and year	Surface modification/Coating	Fabrication method	Release profiles
Fielding et al. (2012)	Ag and Sr doped HA coatings	Plasma spray	Zero order release over 7 days Ag-HA = Sr/Ag-HA
Cheng et al. (2016)	Ag/Sr loaded nanotubular structures Ti surface	Hydrothermal treatment	Burst release over 4 days
He et al. (2016)	Sr/Ag-containing TiO2 coatings	Magnetron sputtering, Micro-arc oxidation	Zero order release over 28 days
Chen et al. (2017)	Sr-Ag incorporated NT-TiO2	Magnetron sputtering, Hydrothermal treatment	First order release over 60 days NT-Ag = NT-Sr- Ag
Geng et al. (2017)	Sr/Ag-co-incorporated HA on a Ti surface	Hydrothermal treatment	Zero order release over 7 days Ag0.1 = Sr/Ag
Huang et al. (2017)	Ag and Sr-doped HA/TiO2 nanotube bilayer coatings	Electrodeposition method	Zero order release over 14 days
Li et al. (2019)	Sr-AgNPs/PDA (polydopamine) coating Ti	Alkali and heat treatment, PDA treatment	Zero order release over 21 days
Qiao et al. (2019)	Si, Sr, Ag co-doped HA/TiO2 coating	Electrodeposition	Zero order release over 14 days AgHA = SSAgHA
Pan et al. (2020)	Sr/Ag-incorporated nanotubes	Hydrothermal treatment, dipping	Burst release over 2 days
van Hengel et al. (2020)	AgNP/Sr functionalized PEO-treated implants	Plasma electrolytic oxidation (PEO)	Burst release over 4 days PT-Sr > PT-AgSr PT-Ag > PT-AgSr
Zhang et al. (2020)	Sr-Ag co-substituted fluorohydroxyapatite nanopillars	Electrolytic deposition	Zero order release over 14 days
Okuzu et al. (2021)	CaSrAg-treated Ti	Alkali heat treatment	Did not evaluate the release profile
Wang et al. (2021a)	silk fibroin/Ag co-functionalized Sr-loaded titanium dioxide nanotubes (SFAgSTN)	Hydrothermal treatment, PDA treatment, layer-by-layer self-assembly	First order release over 14 days AgSTN > SFAgSTN
Zhang et al. (2021)	Sr/Ag-containing TiO_2_ coating	Micro-arc oxidation	Burst release over 1 day
Huang et al. (2022)	Ag-doped SrHA (SrAgHA)/graphene oxide (GO) composite coatings	Electrodeposition	Zero order release over 14 days SrAgHA = SrAgHA/GO
Yao et al. (2022)	Sr/Ag-functionalized hierarchical micro/nano-Ti	Alkali heat treatment, Deposition	Zero order release over 21 days AH-Ti/Ag = AH-Ti/Ag/Sr AH-Ti/Sr = AH-Ti/Ag/Sr
Bian et al. (2023)	HA coating doped with multiple ions (Sr/Ag/Mn) (SrMnAgHA coating)	Electrodeposition	First order release over 14 days

### 3.3 Biocompatibility testing

Before specific antimicrobial and osteogenic testing, all studies but one (Li et al., 2019) tested their surfaces for basic biocompatibility (Table 3). They all used at least two different tests, combining tests for metabolic activity (e.g., MTT assay, CCK-8 assay) with microscopic observations (e.g., CLSM, SEM). Biocompatibility could be verified for all surfaces with combined Ag/Sr coating. Interestingly, compared to Sr only coatings, six of studies showed similar biocompatibility (Fielding et al., 2012; He et al., 2016; Pan et al., 2020; van Hengel et al., 2020; Okuzu et al., 2021; Yao et al., 2022), whereas three studies showed lower (Geng et al., 2017; Zhang et al., 2021; Huang et al., 2022) and one study higher biocompatibility (Wang et al., 2021a). In contrast, compared to Ag only coatings, only one study showed similar biocompatibility (Chen et al., 2017), whereas biocompatibility of eight studies increased (Fielding et al., 2012; Geng et al., 2017; Qiao et al., 2019; van Hengel et al., 2020; Zhang et al., 2020; Wang et al., 2021a; Yao et al., 2022; Bian et al., 2023). The other studies did not compare biocompatibility of these different coatings.

**TABLE 3:** Biocompatibility of functionalized Ti surfaces.

Author and year	Biocompatibility assays	Biocompatibility results
Fielding et al. (2012)	Cell morphology (FESEM)	Sr/Ag-HA = Sr-HA > Ag-HA
	Cell proliferation (MTT assay)	
Cheng et al. (2016)	Cell morphology and adhesion (Fluorescence imaging)	NT-Ag-Sr is biocompatible
	Cell proliferation (CCK-8 assay)	
	Cell migration (Transwell assay)	
He et al. (2016)	Cell adhesion and spreading (CLSM)	M-Sr = M-Sr/Ag
	Cell viability and proliferation (Live/Dead staining, CLSM, MTT assay)	
	Cell morphology (FESEM)	
Chen et al. (2017)	Cell adhesion and morphology (CLSM)	NT-Ag = NT-Sr-Ag
	Cell viability (Live/Dead staining, MTT assay)	
Geng et al. (2017)	Cell morphology (SEM)	Sr > Sr/Ag > Ag
	Cell proliferation (MTT assay)	
	Cell adhesion and distribution (Fluorescence microscopy)	
Huang et al. (2017)	Cell morphology (SEM)	HA = SrAgHA
	Cell proliferation (MTT assay)	
Li et al. (2019)	No biocompatibility testing	
Qiao et al. (2019)	Cell adhesion and proliferation (MTT assay, FESEM, Fluroscence imaging)	SSAgHA > AgHA
	Cell viability (Live/Dead staining)	
Pan et al. (2020)	Cell adhesion and distribution (Fluorescence microscopy)	TNT-Sr/Ag = TNT-Sr
	Cell proliferation (CCK-8 assay)	
van Hengel et al. (2020)	Cell viability (Presto blue assay)	PT-AgSr = PT-Sr > PT-AgSr
	Cell morphology (SEM)	
Zhang et al. (2020)	Cell adhesion (fluorescence microscopy)	SrAgFHA > FHA > AgHA
	Cell morphology (SEM and LSCM)	
	Cell proliferation (MTT assay)	
Okuzu et al. (2021)	Cell viability (XTT assay)	CaSrAg-Ti = CaSr-Ti
	Cell morphology (SEM)	
Wang et al. (2021a)	Cell adhesion (SEM)	SFAgSTN > STN > AgSTN
	Cytotoxicity (LDH assay)	
	Cell proliferation (CCK-8 assay)	
Zhang et al. (2021)	Cell adhesion (fluorescence microscopy)	Sr > Sr/Ag
	Cell morphology (fluorescence microscopy)	
	Cell proliferation (MTT assay)	
Huang et al. (2022)	Cell adhesion and morphology (SEM and LSCM)	SrAgHA/GO = SrHA/GO > SrHA > SrAgHA
	Cell attachment (Fluorescence staining)	
	Cell viability (Live/Dead staining)	
	Cell proliferation (CCK-8 assay)	
Yao et al. (2022)	Cell morphology (FESEM)	AH-Ti/Ag/Sr = AH-Ti/Sr > AH-Ti/Ag
	Cell viability (CCK-8 assay)	
Bian et al. (2023)	Cell adhesion (CLSM)	MnSrAgHA > SrAgHA = HA > AgHA
	Cell toxicity (LDH assay)	
	Cell proliferation (CCK-8 assay)	

### 3.4 Antimicrobial evaluation

Functionalized Ti surfaces were tested for their antibacterial properties using different strains of bacteria and antibacterial assays (Table 4). Eight included studies used one single bacterial species to test their antibacterial efficacy (Fielding et al., 2012; Chen et al., 2017; Huang et al., 2017; Qiao et al., 2019; Pan et al., 2020; van Hengel et al., 2020; Zhang et al., 2021; Bian et al., 2023), while the remaining studies have used more than one type of bacteria (Cheng et al., 2016; He et al., 2016; Geng et al., 2017; Li et al., 2019; Zhang et al., 2020; Wang et al., 2021a; Okuzu et al., 2021; Huang et al., 2022; Yao et al., 2022). The most common used species were *Staphylococcus aureus* (*S. aureus*) representing Gram-positive bacteria and *Escherichia coli* (*E. coli*) representing Gram-negative bacteria. In detail, 6 studies have used only *S. aureus,* one study only *E. coli* and other 9 studies have used both species for the assessment of antibacterial activity. Consequently, only one included study examined the antimicrobial effect of Ti surface using Gram-negative *Pseudomonas aeruginosa* (*P. aeruginosa*) (Fielding et al., 2012).

**TABLE 4:** Antimicrobial properties of functionalized Ti surfaces.

Author and year	Bacteria tested	Antibacterial assay/culture conditions	Antibacterial intergroup comparison (antibacterial rate)	Antibacterial results in Sr/Ag groups
Fielding et al. (2012)	*P. aeruginosa*	Microscopy (fluorescence, live/dead)	Sr/Ag-HA ∼^a^ Ag-HA > Sr-HA ∼ HA	Almost complete eradication of the bacteria (qualitatively)
		24 h planktonic culture		
Cheng et al. (2016)	*MRSA* ^b^, *MSSA* ^c^, *E. coli*	Agar plate based assay (ZOI), Microscopy (SEM)	NT–Ag/Sr > TiO2-NT ∼ Ti	Shrinkage of bacteria and a clear inhibition zone
		24 h planktonic culture, adhesion and biofilm formation		
He et al. (2016)	*S. aureus, E. coli*	Agar plate based assay (CFU), Microscopy (fluorescence, FE-SEM)	M-Sr/Ag0.83 > M-Sr/Ag0.40 > M-Ti and M-Sr	->95% of the bacteria were killed after 6 h (CFU)
		1, 7, 14, 21 and 28 days planktonic culture, adhesion and biofilm formation		-Few or no live bacteria after 1 day (microscopy)
Chen et al. (2017)	*S. aureus*	Agar plate based assay (CFU)	NT-Ag ∼ NT-Sr-Ag	Antibacterial rate:
		12 h up to 60 days planktonic culture		-After 1–10days:100%
				-After 60 days: 84%
Geng et al. (2017)	*S. aureus, E. coli*	Agar plate based assay (ZOI, CFU), Microscopy (SEM, TEM)	Ag ∼ Sr/Ag > Sr ∼ HA ∼ Ti (no antibacterial effect)	- Clear inhibition zone after 24 h
		24 h planktonic culture, adhesion and biofilm formation		-Ruptured bacterial membrane
				−100%–99% reduction in CFU
Huang et al. (2017)	*S. aureus*	Agar plate based assay (CFU, ZOI), Microscopy (SEM, EDS)	CFU reduction: SrAgHA (100%) > Ag/Ha (99.7%) > SrHA (37.2%) > CP-Ti	-Excellent antibacterial effect in all timepoints,
		24h, 36 h or 48 h planktonic culture	ZOI: SrAgHA (25 mm) > Ag/Ha (20 mm) > SrHA ∼ HA (no inhibition)	−100% CFU reduction
Li et al. (2019)	*E. coli, S. aureus*	Agar plate based assay (CFU), Microscopy (SEM)	AH-Sr-AgNPs > Ti	-Round shape bacteria with less pili
		24 h planktonic culture, adhesion and biofilm formation		-Antibacterial efficacy up to 98.66% ± 4.24% (*E. coli*) and 98.17% ± 2.31% (*S. aureus*)
Qiao et al. (2019)	*S. aureus*	Agar plate based assay (ZOI, CFU), 24 h, 36 h planktonic culture	ZOI: AgHA ∼ SSAgHA ∼ SrAgHA > HA (no effect)	-Excellent antibacterial ability,
			CFU reduction of adherent bacteria: AgHA (100%) > SSAgHA (99.5%) > SrAgHA (99.2%)	−99.5% CFU reduction in SSAgHA group
Pan et al. (2020)	*E. coli*	Agar plate based assay (CFU), Microscopy (SEM)	CFU reduction: TNT-Sr/Ag (almost no colonies) > TNT-Sr ∼ TNT > Ti	Good bactericidal properties
		2h and 24 h planktonic culture, adhesion and biofilm formation		
van Hengel et al. (2020)	*MRSA*	Agar plate based assay (ZOI, CFU), *ex vivo* 24h, 48 h planktonic culture, adhesion and biofilm formation	ZOI: PT-Ag/Sr > PT-Ag > PT- Sr ∼ PT ∼ non treated (no inhibition zone)	-Enhanced zone of inhibition
				-No bacterial attachement after 24 h (100%)
				-Compelete eradication of all non-adherent bacteria
Zhang et al. (2020)	*E. coli, S. aureus*	Agar plate based assay (ZOI), General growth analysis (OD), Microscopy (CLSM, live/dead)	SrAgFHA ∼ AgFHA > FHA (no inhibition zone)	>95% Antibacterial rate
		12h and 24 h planktonic culture, adhesion and biofilm formation		
Okuzu et al. (2021)	*E. coli, MSSA*	Agar plate based assay (CFU), Microscopy (fluorescence, SEM)	CFU reduction: CaSrAg-Ti > CaSr-Ti > commercially pure Ti	−95%–99% antibacterial efficay
		24 h planktonic culture, adhesion and biofilm formation		-Shrinked bacteria (SEM)
				-No apparent rupture in bacterial membrane
Wang et al. (2021a)	*S. aureus, E. coli*	General growth analysis (turbity), Agar plate based assay (ZOI, CFU)	Day 1: AgSTN ∼ SFAgSTN > STN	Antibacterial rate of SFAgSTN:
		1, 3, 5 days planktonic culture	Day5: SFAgSTN > AgSTN > STN	−100% after 1 day
				−70% after 5 days
Zhang et al. (2021)	*S. aureus,*	Agar plate based assay (ZOI, CFU), Microscopy (SEM)	Sr/Ag0.34 > Sr/Ag0.17 > Sr/Ag0.08 > Sr/Ag0.04 > Sr/Ag0 (no inhibition zone) (short term)	Long term antibacterial rate (30 days):
		24 h to 30 days planktonic culture, adhesion and biofilm formation		−100% in Sr/Ag0.17, Sr/Ag0.34 without PBS immersion
				−77.60% ± 3.39% in Sr/Ag0.17% and 87.04% ± 1.77% in Sr/Ag0.34 with PBS immersion
Huang et al. (2022)	*S. aureus, E. coli*	Agar plate based assay (CFU), General growth analysis (OD, turbity), Microscopy (CLSM, live/dead, SEM)	SrAgHA/GO ∼ SrAgHA (100%)> SrHA/GO	In SrAgHA and SrAgHA/GO groups:
		24 h planktonic culture, adhesion and biofilm formation		-Irregular and broken morphologies
				-No living adherent bacteria
Yao et al. (2022)	*S. aureus, E. coli*	Agar plate based assay (CFU), Microscopy (fluorescence)	AH-Ti/Ag/Sr ∼ AH-Ti/Ag > AH-Ti/Sr > AH-Ti	Dead percentage of the total bacteria in AH-Ti/Ag/Sr 93.28% (S,aureus), and 87.62% (E.coli)
		24 h planktonic culture, adhesion and biofilm formation		
Bian et al. (2023)	*S. aureus,*	Agar plate based assay (ZOI, CFU), Microscopy (fluorescence)	AgMnSrHA (100%) > MnSrHA (20%) > SrHA ∼ HA (no antibacterial effect) (24 h)	Bactericidal rate of AgMnSrHA:
		1, 3 days planktonic culture, adhesion and biofilm formation		−100% after 1 day
				−80% after 3 days

^a^ Refers to no significant difference.

^b^ *MRSA*: Methicillin-resistant *Staphylococcus aureus*.

^c^ *MSSA*: Methicillin-sensitive *Staphylococcus aureus*.

There are three categories of antibacterial assays that were used in the selected studies: microscopy methods (fluorescence or scanning electron microscope (SEM)) (13 publications); agar plate-based assays including zone of inhibition (ZOI) and colony forming units counting (CFU) (16 publications); and general growth analysis including the turbidity and the optical density measurement (OD) (3 publications). Out of the 17 included studies, two studies used only single method for the assessment (Fielding et al., 2012; Chen et al., 2017), and the remaining used two or more assays. These assays were applied to planktonic cultures in all studies, but further twelve studies analyzed additionally the adhesion and formation of biofilms (Cheng et al., 2016; He et al., 2016; Geng et al., 2017; Li et al., 2019; Pan et al., 2020; van Hengel et al., 2020; Zhang et al., 2020; Okuzu et al., 2021; Zhang et al., 2021; Huang et al., 2022; Yao et al., 2022; Bian et al., 2023). However, mostly attachment and biofilm formation were only evaluated qualitatively. Quantitative analysis of attached bacteria or formed biofilm was evaluated only in four studies using live/dead staining and microscopic analysis (Zhang et al., 2020; Huang et al., 2022; Yao et al., 2022; Bian et al., 2023). The general antibacterial activity was quantitatively measured in all studies except one, which qualitatively examined the antibacterial effects using live/dead fluorescence images (Fielding et al., 2012). Although all of the included studies evaluated the antibacterial effects over a period of 24 h, long-term antibacterial effects was additionally examined in four studies up to 5, 28, 30 and 60 days (He et al., 2016; Chen et al., 2017; Wang et al., 2021a; Zhang et al., 2021).

Table 4 presents summary of antibacterial efficacy of Sr/Ag functionalized Ti surfaces. All studies showed antibacterial effect against tested bacterial strains. The short-term (up to 24 h) antibacterial rate was reported between 95% and 100% in 13 studies (He et al., 2016; Chen et al., 2017; Geng et al., 2017; Huang et al., 2017; Li et al., 2019; Qiao et al., 2019; van Hengel et al., 2020; Zhang et al., 2020; Wang et al., 2021a; Okuzu et al., 2021; Zhang et al., 2021; Huang et al., 2022; Bian et al., 2023). Two studies did not quantitatively report the antibacterial efficacy but showed nearly a complete eradication of bacteria in flurescence imaging (qualitatively examined) and agar plates (no report of counting, but stated as significant), respectively (Fielding et al., 2012; Pan et al., 2020). Cheng, et al. also reported their antibacterial activity by measuring the inhibition zone which was reported 0 cm in case of Ti and TiO_2_–NT groups, and between 1.52 ± 0.10 cm to 1.94 ± 0.21 cm in groups containing Sr/Ag (Cheng et al., 2016). Further, Yao et al. demonstrated 93.28% and 87.62% dead percentage of the total bacteria in alkali heat treated-Ti/Ag/Sr groups against *S. aureus* and *E. coli* subsequently (Yao et al., 2022). By comparing the long-term antibacterial activity (≥5 days), the effect was reduced by time in all studies which have exmained this effect in a longer period of time. Chen, et al. showed reduced antibacterial effect from 100% to 84% after 60 days (Chen et al., 2017). However, He, et al. revealed more severe reduction to 40% after even 28 days (He et al., 2016). Testing silk fibroin/Ag co-functionalized Sr titanate nanotubes showed a 30% reduction in antibacterial efficacy after 5 days (Wang et al., 2021a). Interestingly, immersing Sr/Ag coated Ti surfaces in PBS showed reduced antibacterial activity in the long term comparing the surface without PBS immersion (Zhang et al., 2021). Furthermode, five studies compared the antibacterial potential of final coating containing Sr/Ag with only Ag functionalized groups (without Sr, but with or without other chemicals) (Huang et al., 2017; Qiao et al., 2019; van Hengel et al., 2020; Zhang et al., 2020; Yao et al., 2022). Out of these publications two of them showed enhanced antibacterial effect in the Sr/Ag functionalized groups in comparison to Ag groups without Sr addition (Huang et al., 2017; van Hengel et al., 2020). Remaining studies have shown that the combination of Sr and Ag had no beneficial effects on antibacterial efficiency of Ag (Qiao et al., 2019; Zhang et al., 2020; Yao et al., 2022). Additionally, six studies also evaluated the antibacterial effect of Sr groups (without Ag). Out of these, four showed limited or no antibacterial effect (Chen et al., 2017; Pan et al., 2020; Okuzu et al., 2021; Zhang et al., 2021), whereas two studies demonstrated antibacterial effects following the application of Sr functionalized Ti groups (Huang et al., 2017; van Hengel et al., 2020).

### 3.5 Osteogenic characteristics

Similar to antibacterial testing, assessment of osteogenic characteristics was done using different cell types and methods. Table 5 shows a summary of the included studies regarding cell type used, methods used for analyzing the expression of osteoblastic phenotype, osteogenic markers, and intergroup comparison. Commonly used cell systems in the studies comprised osteoblast-like cell lines and stem cells. Among the included studies, 16 of them have used cell lines: 14 used MC3T3-E1 as a mouse non-transformed cell line; one publication used MG63 (human osteosarcoma cells) (Geng et al., 2017) and one study human fetal osteoblastic cells (hFOB) (Fielding et al., 2012) (Table 5). Exceptionally, Okuzu, et al. tested osteogenic activity of surfaces using rat bone marrow stromal cells (BMSCs) (Okuzu et al., 2021). As defined by the inclusion criteria, all included investigations observed stimulation towards osteogenic differentiation. Depending on the stages of differentiation and coatings, osteogenicity was triggered with the addition of certain factors or as an effect of coating itself or the combination of both. For example, six studies mentioned using beta-glycerol phosphate/ascorbic acid and dexamethasone or their combination for osteogenic induction (Cheng et al., 2016; He et al., 2016; Chen et al., 2017; van Hengel et al., 2020; Okuzu et al., 2021; Yao et al., 2022). Remaining studies either checked the osteogenic differentiation without the addition of extra osteogenic triggers or did not report in their study design. In case of BMSC, osteogenic induction medium has been implemented to activate osteogenic differentiation (Okuzu et al., 2021).

**TABLE 5:** Osteogenic outcome of functionalized Ti surfaces.

Author and year	Cells tested	Assays detecting expression of osteoblastic phenotype	Osteogenic markers	Osteogenic intergroup comparison
Fielding et al. (2012)	hFOB	Immunohistochemistry	ALP	Sr/Ag-HA ∼ Sr-HA > HA > Ag-HA (qualitative)
Cheng et al. (2016)	MC3T3-E1	ARS staining, Osteogenic gene expression (RT-PCR)	ECM Calcium deposition (21 days), RUNX2, OCN, ALP, Col-I, OPG (14 days)	Mineralization, RUNX2, OCN: NT40–Ag_1.5_Sr_3_ ∼ NT40–Ag_2.0_Sr_3_ > NT10–Ag_1.5_Sr_3_ ∼NT10–Ag_2.0_Sr_3_ > TiO2-NT ∼ Ti
				ALP, Col-I: NT40–Ag_1.5_Sr_3_ ∼ NT40–Ag_2.0_Sr_3_ ∼ NT10–Ag_1.5_Sr_3_ ∼ NT10–Ag_2.0_Sr_3_ > TiO2-NT ∼ Ti
				OPG: not reported
He et al. (2016)	MC3T3-E1	Direct Red 80, ARS staining	Col (7days), ECM calcium deposition (7, 14 days)	Col: M-Sr/Ag0.40 > M-Sr > M-Ag0.40 >∼ M-Ti
				Day 7 mineralization: no significant difference!
				Day 14 ECM mineralization: M-Sr/Ag0.40 > M-Ag0.40 >∼ M-Ti AND M-Sr/Ag0.83 ∼ M-Ag0.83 >∼ M-Ti
Chen et al. (2017)	MC3T3-E1	ALP Kit, Direct Red 80 staining, ARS staining	ALP (3, 7 days), Col (7, 14 days), ECM calcium deposition (14, 21 days)	Day 7 ALP: NT-2Sr-Ag > NT-3Sr-Ag > NT-Ag ∼ pure Ti
				Day 14 Col: NT-3Sr-Ag > NT-1Sr-Ag > pure Ti
				Day 14 and 21 mineralization: NT-2Sr-Ag > NT-Ag ∼ pure Ti (Col day 7, 14)
Geng et al. (2017)	MG-63	ELISA kit, Osteogenic gene expression (RT-PCR)	ALP OCN, RUNX2, (3, 7, 14 days)	Sr > Sr/Ag > HA > Ti > Ag
Huang et al. (2017)	MC3T3-E1	ALP kit, OCN ELISA kit, fluorescence staining	ALP (7, 14 days), OCN (14 days)	Day 7: no significant difference!
				Day 14: SrAgHA ∼ Ha > Ti
Li et al. (2019)	MC3T3-E1	Osteogenic gene expression (RT-PCR), ARS staining	ALP, Runx2, COL-1 (7 days), Calcium deposition (21 days)	AH-Sr-AgNPs + MO > control + MO
				Monoculture (mineralization): AH-Sr-AgNPs > AH-Sr > AH-AgNPs ∼> Ti
				Coculture (minerlization): AH-Sr-AgNPs > AH-AgNPs > AH-Sr ∼ Ti
Qiao et al. (2019)	MC3T3-E1	Osteogenic gene expression (RT-PCR)	ALP, BMP-2, RUNX-2, OCN, OPN, Col-1 (14 days)	SSAgHA > HA > Ti
Pan et al. (2020)	MC3T3-E1	ALP and OCN kit, ARS staining	ALP, OCN (1, 3 days), ECM calcium deposition (7, 21 days)	Day3: TNT-Sr/Ag ∼ TNT-Sr > TNT > Ti
				Day 21: TNT-Sr/Ag ∼ TNT-Sr > TNT > Ti
van Hengel et al. (2020)	MC3T3-E1	ALP kit	ALP	Day 11: PT-Sr ∼ PT-Ag Sr > PT-Ag > NT
Zhang et al. (2020)	MC3T3-E1	ALP kit, OCN ELISA kit	ALP (1, 7, 14 days), OCN (14 days)	Day 7 and 14, ALP: SrAgFHA ∼ FHA > AgFHA > Ti
				OCN: SrAgFHA > FHA > AgFHA > Ti (OCN)
Okuzu et al. (2021)	BMSCs	Osteogenic gene expression (RT-qPCR), ALP kit	ALP, RUNX2, OCN, OPN (7, 14 days)	Day 14, Runx2,ALP: CaSrAg-Ti > CaSr-Ti > Ti
				Day 7, OCN: CaSrAg-Ti > Ti
				Day14, OCN: CaSr-Ti > Ti
Wang et al. (2021a)	MC3T3-E1	ALP kit, Pro-Col I and Osteocalcin ELISA kit	ALP (3, 7, 14 days), OCN, Col1 (14 days)	Day7 and 14, ALP: SFAgSTN > STN > AgSTN ∼ Ti
				OCN, Col: SFAgSTN ∼ STN > AgSTN ∼ Ti
Zhang et al. (2021)	MC3T3-E1	ALP kit	ALP (7 days)	Sr90/Ag0.17 > Sr0/Ag0.17 (before and after PBS immserion)
Huang et al. (2022)	MC3T3-E1	ALP, RUN and Col I staining	ALP (qualitative), Col I, RUN (qualitative), (7, 14 days)	Day 14, Col: SrHA/GO > SrAgHA/GO ∼ SrHA > SrAgHA ∼ HA > Ti
Yao et al. (2022)	MC3T3-E1	ELISA kit, Osteogenic gene expression (RT-qPCR), ARS staining	ALP (4, 7 days), OCN (7, 14 days), RUNX2, OPN (7days), ECM calcium deposition (14, 21 days)	AH-Ti/Ag/Sr ∼ AH-Ti/Sr > AH-Ti/Ag ∼ AH-Ti
Bian et al. (2023)	MC3T3-E1	ALP kit	ALP (1, 7, 14 days)	Day 14, 7: MnAgHA ∼ SrAgHA > HA > AgHA > Ti

Different *in vitro* methods were used to estimate the expression of the osteoblastic phenotype. These methods include reverse transcriptase PCR (RT-PCR) for the detection of osteogenic transcripts, immunochemistry methods like enzyme-linked immunosorbent assay (ELISA) and immunofluorescence microscopy for secreted protein markers, colorimetric assays for osteogenic enzymes, and direct staining of matrix components. ALP activity was the most frequently studied osteogenic marker in the included studies and has been reported in 16 studies (Table 5). ALP activity was either detected qualitatively by staining or determined quantitatively by colorimetric analysis or RT-PCR. Six studies have used alizarin red staining as a marker for ECM mineralization (Cheng et al., 2016; He et al., 2016; Chen et al., 2017; Li et al., 2019; Pan et al., 2020; Yao et al., 2022), while Sirius Red staining (Direct Red 80) as a marker of collagen secretion was used in three studies (He et al., 2016; Chen et al., 2017; Huang et al., 2022). Furthermore, six studies have employed RT-PCR to detect the expression of osteogenic markers, such as ALP, Runx2, OCN, Col-I, and OPN (Cheng et al., 2016; Geng et al., 2017; Li et al., 2019; Qiao et al., 2019; Okuzu et al., 2021; Yao et al., 2022). Within each study, several osteogenesis markers were used to confirm osteogenic differentiation: seven used two osteogenic markers and ten studies explored three or more osteogenic markers (Table 5).

To evaluate the biological effects of coated Ti in a more complex environment, an additional co-culture of two cell types or cells and bacteria was performed in two studies. Chen et al. used supernatant of MC3T3-E1 culture medium to evaluate its effect on human endothelial cell line (ECs) and *in vitro* angiogenesis (Chen et al., 2017). Similarly, Li et al. implemented MC3T3-E1 in an indirect co-culture with supernatants of Raw 264.7 (murine macrophage cell line) cultured on different Ti substrates (Li et al., 2019). Their results showed more elongated cells and expression of osteogenic markers in stimulated groups by the supernatants indicating early bone healing facilitation by regulating macrophage polarization (Li et al., 2019). Additionally, they have compared the mineralization rate with and without the presence of bacteria after 21 days. Mineralization value in all groups which have been used in co-culture setups was lower than those in mono-culture setup (Li et al., 2019).

The *in vivo* osteoinductive potential of combined Sr/Ag-modified surfaces was investigated in four included studies (Li et al., 2019; Zhang et al., 2020; Wang et al., 2021a; Yao et al., 2022). Among them, one has used rabbit’s maxila as implant insertion location (Yao et al., 2022), while others have used rabbit’s femoral defect model for their *in vivo* evaluation. Li, et al. have contaminated the implants with *S. aureus* prior implantation (Li et al., 2019). Animals were monitored between 4 weeks and 8 weeks and subsequently sacrificed for further analysis. All studies used histopathological analysis of surrounding tissue. A variety of histological staining techniques such as Hematoxyline and eosin (HE), Immunohistochemistry, Van Gieson and Masson’s Trichrome were used to determine the new bone formation, inflammatory cell infiltration, collagen fibers and fibrosis. Using micro-computed tomography (µCT) in two studies, it was possible to quantify the amount of newly formed bone surrounding implants based on the bone-to-tissue volume ratio (BV/TV) (Li et al., 2019; Yao et al., 2022). As an additional analysis, the study by Li et al. injected ARS and calcein intramuscularly to evaluate dynamic osteogenesis process and bone growth pattern (Li et al., 2019).

In all studies, Sr/Ag modified Ti surfaces showed an increased osteogenic effect in comparison to one or more than one control groups (Ti, Ag-Ti, Sr-Ti) (Table 5). As most studies applied not only Sr but also different surface structuring and/or soluble factors to promote osteogenic differentiation, a direct comparison of efficiency was difficult. Overall, six studies reported a mild increase in osteogenic differentiation of up to 20% in the Ag/Sr groups compared to the unmodified controls (Chen et al., 2017; van Hengel et al., 2020; Zhang et al., 2020; Wang et al., 2021a; Huang et al., 2022; Bian et al., 2023). A medium increased differentiation of approx. 30%–40% was reported in further five studies (He et al., 2016; Li et al., 2019; Pan et al., 2020; Zhang et al., 2020; Okuzu et al., 2021), whereas four studies detected a comparable strong differentiation increase of approx. 50% (Cheng et al., 2016; Huang et al., 2017; Qiao et al., 2019; Yao et al., 2022). The strongest increase in osteogenic differentiation with 60%–70% was observed by Geng et al. in hydrothermal silver-containing calcium-phosphate coatings with strontium as binary dopant (Geng et al., 2017). Additionally, ten studies have compared the effect of Sr/Ag modified surfaces (alone or in combination with other modifications) with Sr functionalized control groups (Fielding et al., 2012; He et al., 2016; Geng et al., 2017; Li et al., 2019; Pan et al., 2020; van Hengel et al., 2020; Wang et al., 2021a; Okuzu et al., 2021; Huang et al., 2022; Yao et al., 2022). Four of them showed more osteogenic effect in Sr/Ag groups (with or without extra biofunctional aganets) in comparison to Sr alone groups (He et al., 2016; Li et al., 2019; Wang et al., 2021a; Okuzu et al., 2021). In detail, He, et al. reported more collagen secretion in M-Sr/Ag 0.40 group in compare with M-Sr at day 7. Although, ECM mineralization did not show a similar effect (He et al., 2016). On the other hand, Li, et al. showed enhanced mineralization in AH-Sr-AgNPs group in comparison with AH-Sr in both mono and co-culture conditions (Li et al., 2019). Additionally, Okuzu, et al. revealed higher Runx2 and ALP expression in CaSrAg-Ti in compare with CaSr-Ti group (Okuzu et al., 2021). Synergistic osteogenic effects of Sr with SF showed higher ALP secretion in nano-silver-silk fibroin Sr-loaded TN in comprae to Sr-loaded TN (Wang et al., 2021a). The heterogeneous measurement approaches employed across the included studies made quantification and comparison of osteogenic effects difficult in the context of our systematic review. In particular, there were differences in selection of osteogenic markers, the timing of assessments and variability in reporting.

### 3.6 Risk of bias assessment

**FIGURE 2:**
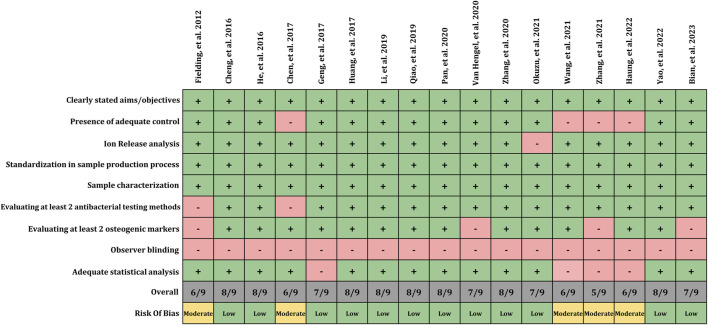
Risk of bias assessment.

The risk of bias assessment among the included studies is illustrated in Figure 2. All included studies (100%) clearly defined the aim and objectives, which aid in ease of assessment of methods and conclusion. Moreover, it facilitates readers and researchers in the decision-making process. 76% of included study compared test group with adequate controls (unmodified surfaces) thus facilitating effective comparison and interpretation of results with the standard. Whereas, remaining 24% of included study did not report or utilize the control group. Ion release from the surfaces was measured in 94% of studies. It is noteworthy to report that 100% of included studies presented standardization in sample processing and sample characterization. Majority of the included studies (88%) utilize at least two tests for antibacterial assessment. Assessment of antibacterial effects with minimum two test is significant as it will help to report the confirmative analysis of one test with other. Thus, indicating a low risk of bias in assessing the antibacterial effect of proposed implant material. Whereas remaining 12% of included studies used only one test for its assessment. Similarly, findings were observed in terms of osteogenic assessment, wherein majority of included studies (76%) reported or utilize at least 2 different methods to evaluate the osteogenic potential whereas remaining 24% included studies failed to report two methods. No study reported the blinding of the observer. This would have allowed to avoid bias while assessing and interpretation of the data among the test and control group. 76% of studies presented adequate statistical analysis. Overall, 12 included studies reported a low risk of bias, and 5 studies reported a moderated risk of bias.

## 4 Discussion

Simultaneous dual-functionality towards antibacterial and osteoinductive effect can be achieved on Ti implant surfaces by combination of multiple functional elements. However, striking an optimal balance between enhancing osseointegration and minimizing infections remains challenging. This is due as most of bactericidal materials do not exhibit acceptable antibacterial properties within the cytocompatible range, and high amounts of osteoinductive materials can be too toxic for the cells as well. Moreover, the functionality of these materials can be influenced by factors such as coating strategy, functional element concentration, release kinetics, and the specific chemical properties. The present systematic review provides a comprehensive PICOS-based analysis of the effectiveness of Ti surfaces modified with combinations of Sr as an osteoinductive agent and Ag as an antibacterial agent. The included studies used different methods tailored to the metallic titanium surface that led to a superficial modification of Ti implants with Sr/Ag. Applying different methods, like plasma immersion ion implantation or sol-gel chemistry, could have led to a modification of also deeper material layers or a transferability to other, more temperature sensitive materials, respectively (Xue et al., 2020). The release of ions from these surfaces mainly followed a zero or first order release, rather than a burst release, which is promising for future application as it guarantees a longer lasting effect. Also, the type of incorporated ions (individual or combined) seems not to affect the release properties, even if a final conclusion would require additional analyses. All studies reported basic biocompatibility of the combined Ag/Sr coating. The biocompatibility was mainly comparable to surfaces with Sr only and mostly higher than for surfaces with Ag only. Ag’s application on tissue cells commonlly causes cytotoxic reactions not only for implants but also other biomaterials including impairment of membrane integrity, oxidative stress and DNA damage (Abram and Fromm, 2020; Liu et al., 2023). However, incubation time and concentration strongly affect cytotoxicity level. According to Pauksch. et al., hMSC viability was reduced significantly by Ag application after 21 days, despite no cytotoxic effects observed after 2 h and 7 days (Pauksch et al., 2014). The increased biocompatibility of the Ag/Sr combined coatings indicates that the promoting effect of Sr could counterbalance the cytotoxic effects of Ag at least to a certain extent. However, the mechanism behind this observation is unclear and would need to be analyzed in future studies.

Regarding the major focus of this work, all studies included in this review reported dual-functionality, enhanced antibacterial and osteogenic effects, through physical, chemical, and biological modifications. To evaluate these antibacterial and osteogenic effects a wide range of standard methods was applied. However, the results of these evaluations were not dependent on the specific methods employed or the number of methods used.

**FIGURE 3:**
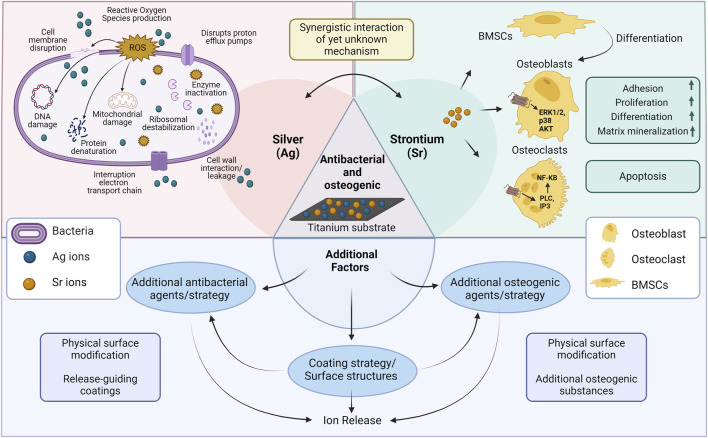
Schematic illustration of possible mechanisms related to antibacterial/osteogenic titanium surfaces in the included studies. Abbreviations: BMSCs, bone marrow stromal cells; ERK1/2, extracellular signal-regulated kinases 1/2; NF-KB, nuclear factor k-light-chain-enhancer of activated B cells (Wang and Yeung, 2017; Marx et al., 2020; Yin et al., 2020; You et al., 2022). Created with BioRender.com.

The antimicrobial effect of the coated Ti surfaces is primarily associated with the presence of Ag, more specifically the released silver ions. As already reviewed and illustrated elsewhere, silver ions are able to adhere to and disrupt the bacterial membrane (Figure 3). By this, they enter the cytoplasm, where they impair multiple cellular structures, enzymes and pathways, e.g., glycolysis or cellular stress response, mainly through the generation of reactive oxygen species, which finally leads to cellular dysfunction and bacterial death (Wang et al., 2021b; Tripathi and Goshisht, 2022). Although, certain studies have utilized additional substances, structures or mechanisms in order to: 1) reduce burst release of Ag resulting in lower cytotoxicity and longer antibacterial effects; 2) synergistically enhance antibacterial effects allowing them to employ these agents at low concentrations to minimize their potential cytotoxicity (Figure 3). For instance, silk fibroin’s cross-linking effect prevents sudden releases of Ag ions and prolongs antibacterial activity (Wang et al., 2021a). A similar effect was obtained by adding Sr and GO to the coating resulting in reduced Ag burst release (Huang et al., 2022). Furthermore, Yao et al. demonstrated that hierarchical micro/nanostructures have the ability to incorporate adequate amounts of nanoparticles for gradual release of metal ions (Yao et al., 2022). Interestingly, two studies could detect antibacterial effects also in Sr groups without Ag, resulting in a synergistic antibacterial effect against *S. aureus* or *MRSA* in the final coating in addition to Ag (Huang et al., 2017; van Hengel et al., 2020). Our recent *in vitro* investigation reported similar findings, where the combination of Sr acetate with Ag nitrate resulted in a synergistic increase in the antibacterial effect against *Aggregatibacter actinomycetemcomitans*. However, so far, the mechanism is unclear (Figure 3) as the synergistic effect might have different mechanism of action in killing bacteria than the individual substances (Parizi et al., 2022). It might be hypothesized that the effect includes changes in environmental factors, like pH or increasing of membrane permeability. Based on the release patterns, there is no indication of an influence of substance release kinetics. Additionally, certain coating structures such as nanoscale surfaces, may exhibit antimicrobial properties (Grischke et al., 2016). In the study by Pan et al., anodization of titanium surfaces increased surface hydrophilicity, preventing non-specific adsorption of bacterial adhesion proteins and reducing bacterial attachment (Pan et al., 2020). A study by Cheng et al., however, found that no ZOI was observed in pure Ti and TiO_2_-nanotubes, indicating no antibacterial activity following nanostructure modifications (Cheng et al., 2016). Thus, Ag appears to play the major role in antibacterial results, but there may also be additional effects resulting from other substances or structures, resulting in synergistic interactions that could be attributed in further studies.

The critical time point for preventing implant infections can vary depending on the type of implant and the specific clinical context in which it is placed. However, in general, the immediate post-surgery period is considered the crucial time period for preventing implant infections. Bacteria can colonize on surface of dental implants within the initial 30 min after implant placement (Fürst et al., 2007). Accordingly, most of the studies included in this review evaluated the antimicrobial effects of functionalized surfaces at an early stage (up to 24 h). At earlier time points, immediate release of Ag ions showed a stronger antibacterial effect due to higher ion concentrations. As time progresses, Ag ion release may decrease, reducing antibacterial activity (He et al., 2016; Chen et al., 2017; Wang et al., 2021a). Successful clinical application of these surface coatings would require more detailed understanding of durability and sustainability of the antibacterial properties both the in short- and long-term setups, which should be adressesd in further studies. The effect of antibacterial agents also varied depending on the sensitivity of different bacteria. Accordingly, *E. coli* showed more sensitivity to Ag’s bactricidal effects, whereas *S. aureus* required a longer time or higher concentration to reach the same level of inhibition (He et al., 2016; Geng et al., 2017; Okuzu et al., 2021). In contrast, Yao, et al. showed higher percentage in dead *S. aureus* (93%) in comparison to *E. coli* (88%) (Yao et al., 2022). Similarly, our previous investigation on antibacterial effect of Ag/gold NP against *S. aureus* and *Porphyromonas gingivalis* not only yielded different antibacterial outcomes depending on the bacterial species but also varied according to method of analysis (Doll-Nikutta et al., 2023). Most of the included studies in the current review have used *S. aureus* or *E. coli* or both for bacterial testing which are amongst the most clinical relevant strains for endoprosthetic joint infections (Otto-Lambertz et al., 2017). However, they are not representative of common bacterial species in the oral cavity, so the results from these articles most probably cannot be directly translated to dental implant applications, which account for the highest number of implantations. In line with this, most of the included studies investigated the potential antimicrobial effects on only one or two individual bacterial strains. This is beneficial as using single bacterial strains allows for easier comparison of results across studies and between different antibacterial implant surfaces. Nevertheless, it was found that antimicrobial treatment was less effective against multispecies biofilms that are commonly associated with dental peri-implant infections due to heterogeneity of species (Flemming et al., 2016). Most of the studies considered bacterial attachment and biofilm formation when analyzing antibacterial effectiveness. This is crucial as biofilms are the clinically relevant growth form and at the same time possess inherent resistance to antibacterial strategies (Davies, 2003). However, only some studies analyzed biofilms not only qualitatively but also quantitatively, probably due to limitations in specialized analysis software. In summary, for reliable clinical translation, the selection of bacteria and their growth forms should be compatible with the desired application and reflect clinical infection conditions. This ensures practicality and makes these strategies more transferable to clinical settings.

Besides antibacterial effects, all the included studies showed enhanced expression of at least one osteogenic marker by the application of dual-functionalized surfaces. Considering the variations in osteogenic evaluation methods employed among the included studies, a general overview reveals that Sr/Ag modified titanium surfaces enhance osteogenic characteristics in multiple aspects including cellular functionality, mineralization capacity, ALP and Col-I expression, and osteogenic gene expression. Following cellular interaction with modified surfaces, osteoblasts functionality (adhesion, spreading, migration, proliferation, methabolic activity, viability) was enhanced in all included studies facilitating osteogenic differentiation. Furthermore, most of the coatings enhanced the expression of ALP, an enzyme that reduces the extracellular pyrophosphate concentration which inhibits mineral formation, thereby facilitates mineralization (Vimalraj, 2020). The modified surfaces positively influenced mineralization, promoting the deposition of calcium and phosphate ions essential for the formation of hydroxyapatite crystals (Cheng et al., 2016; He et al., 2016; Chen et al., 2017; Li et al., 2019; Pan et al., 2020; Yao et al., 2022). Additionally, an observed increase in collagen synthesis and expression of osteogenic genes signifies the activation of osteogenic signaling pathways and the promotion of osteoblastic activity, thereby contributing to the enhanced formation of bone tissue (Cheng et al., 2016; He et al., 2016; Chen et al., 2017; Geng et al., 2017; Li et al., 2019; Qiao et al., 2019; Okuzu et al., 2021; Huang et al., 2022; Yao et al., 2022). Based on the *in vitro* and *in vivo* findings presented and illustrated in a recent review, Sr-based biomaterials exhibit enhanced expression of osteogenic markers, reduced bone resorption, enhanced osteoinduction, and improved bone healing at the bone-implant interface mainly by strontium’s binging to the calcium sensing receptor (Figure 3) (Marx et al., 2020). However, it has to be noted that even though multiple studies analyzed the expression of osteogenic genes on RNA level, non of them stepped into the detailed mechanisms of RNA activity regulation. In this regard, RNA modifications or mRNA-based mechanisms could be analyzed in future (Chen et al., 2020; Chen et al., 2022).

It is important to note that this increase in osteogenic properties cannot be solely attributed to Sr, as there may also have been other factors involved (Figure 3): 1) physical surface modifications, 2) the presence of additional osteogenic agents in the coatings or modifications used in the studies, and 3) indirect effect of Ag’s antibacterial effect providing a favorable environment for osteoblasts’ function. Surface roughness and nanostructures can enhance osteoblast interactions with the surface as well as protein absorption resulting in osteoblast maturation, increased bone implant contact (BIC) and better osseointegration rate (Jemat et al., 2015). Osteogenic activity may have been accelerated by these factors working synergistically with Sr. For instance, HA, GO and Mn as well-known osteoinductive materials have synergistically enhanced osteogenic potential of functionalized surfaces (Fielding et al., 2012; Qiao et al., 2019; Zhang et al., 2020; Huang et al., 2022; Bian et al., 2023). Similar results were observed in a review demonstrating that Sr-doped HA and metal-based biomaterials increased osteogenic differentiation and prevented bone loss *in vitro* and *in vivo* respectively (Borciani et al., 2022). Interestingly, three studies (out of four studies which had adequate control groups) have shown that combined treatment of Sr/Ag on a similar surface without extra osteogenic agents also enhances the osteogenic effect compared to Sr alone groups, indicating a synergistic effect of the two elements (He et al., 2016; Li et al., 2019; Okuzu et al., 2021). Depending on the study, corresponding effect has either been attributed directly to osteogenic effect of Ag or indirectly as a result of antibacterial properties of Ag. To be more specific, Ag0.40 modified surfaces showed higher cell proliferation and osteogenic differentiation as compared to control Ti, indicating that adequate levels of released Ag enhance osteogenesis (He et al., 2016). In summary, this review demonstrates that synergistic interactions of antibacterial and osteogenic substances is evident in several studies, however, detailed knowledge on their extent and mechanism is very limited so far and requires more research in future.

A majority of studies have utilized cell lines, 2D culture systems, and monocultures for their evaluations. While these approaches provide valuable insights, they do not adequately reflect the complex interactions between bacteria, tissue and modified surfaces in the clinical situation. This is supported by the results of Li. et al., where Ti-containing Ag groups showed increased mineralization values compared to groups without Ag in co-culture with bacteria. This was attributed to Ag’s antibacterial properties providing a favorable environment for cell differentiation by protecting them from bacterial invasion. Although, mono-culture mineralization rates were lower in Ag-treated groups than in Sr-treated groups (Li et al., 2019). This strengthens the importance to examine Ti surfaces with dual antibacterial and osteogenic properties in a co-culture setup that closely mimics clinical situations to already include the complex bacteria-cell-interactions for *in vitro* analyses. Moreover, it is essential to consider the triangular interactions between bacteria, cells, and the surface when evaluating the antibacterial and osteogenic effects of titanium which may have a significant impact on the final results. Further studies can be conducted using primary cells, co-culture systems, 3D cultures, and *in vivo* models to gain a deeper understanding of the coatings’ efficacy.

Even though this systematic review specifically searched for *in vitro* studies the promising results regarding the dual functionality of Ag/Sr modified titanium surfaces make a consideration of their clinical application worth it. A first point that need to be considered is the expected mechanical stability as all applied functionalizations were superficial coatings (Table 2). Titanium implants, both for dental and orthopedic applications, experience harsh shear stresses during implantation, which often only full material modifications can withstand (Denis et al., 2024). Thus, the developed functionalizations need to be tested for their stability and if not fulfilling these requirements alternative application areas with less shear stress, like dental abutments should be considered. Furthermore, as already addressed in the last paragraph, a validation of these *in vitro* results in more realistic biological setups is required. This should include the interaction between human soft and hard tissue cells with the bacteria, but also the reaction of immune cells. The analyses could be performed in *in vivo* models (Blank et al., 2021) or by using sophisticated 3D *in vitro* models (Ingendoh-Tsakmakidis et al., 2019), if available for the desired application. As all coatings reviewed in this study change surface chemistry and not only physical properties, the *in vitro* and *in vivo* results could finally lead to clinical studies and subsequent medical device approval.

## 5 Conclusion

With regard to the initial PICOS question, incorporating Sr and Ag into Ti implant surface modifications could improve the antibacterial and osteogenic properties of the surface in all included studies. Surface modification with both substances was achieved by different coating strategies that all exhibited comparable release properties and biocompatibility. Antibacterial activity was mainly assessed using *E. coli* and *S. aureus* as model organisms and classical microbiologic and microscopic techniques. The strong antibacterial effect of all surfaces could be mainly attributed to the release of silver ions, even though physical surface structuring and a yet barely studied antibacterial effect of Sr could have contributed as well. Osteogenic properties were characterized using osteoblast-like cell lines or stem cells analyzed with multiple different methods ranging from staining techniques to molecular analyses. By this, increased osteogenic differentiation could be detected, which can be linked to the ability of Sr binding to the Calcium sensing receptor but also to physical surface structuring, additional osteogenic substances as well as surface clearance by antibacterial Ag. Interestingly, some studies could detect a synergistic enhancement of antibacterial and osteogenic properties with Ag/Sr dual-functionalized surfaces. The underlying mechanism is not known so far but would be interesting to address in future research. Further investigations should also validate these results regarding specific bacterial strains, the type of cells used, method of analysis, as well as co-culture or mono-culture setups to account for the need of testing systems close to the natural situation to improve clinical translation. Furthermore, also additional physical factors, which were not part of this review, need to be considered, like mechanical stability of the coatings. Consequently, further *in vitro* and *in vivo* research specifically tailored to the desired clinical application is required to evaluate the potential benefits and limitations of using Sr and Ag in combination for finally improving titanium implants’ biocompatibility and patient treatment.

## Data Availability

The original contributions presented in the study are included in the article/Supplementary Material, further inquiries can be directed to the corresponding authors.
